# Populism in Peru: Populist attitudes and perception of the populist offer and its relationship with political cynicism and attitudes toward democracy

**DOI:** 10.3389/fpsyg.2022.1070609

**Published:** 2022-12-20

**Authors:** Agustin Espinosa, Erika Janos, Manuel Pacheco, Juan Juárez, Hernán Chaparro

**Affiliations:** ^1^Departamento de Psicología, Pontificia Universidad Católica del Perú, Lima, Peru; ^2^Departamento de Humanidades y Ciencias Sociales, Universidad Nacional de San Martín, Tarapoto, Peru; ^3^Facultad de Comunicación, Universidad de Lima, Lima, Peru

**Keywords:** attitudes toward democracy, populist attitudes, political cynicism, perception of populist offer, Peru

## Abstract

Populism is a phenomenon that is gaining attention in Political Psychology. The goal of the current study was to determine the relationship between populist attitudes, based on the populist demand and the perception of the populist offer, and several indices of political cynicism and attitudes toward democracy in Peru. To do this, a quantitative correlational study including 391 participants from diverse Peruvian locations was carried out. Both populist attitudes and critical perception of the populist offer are found to be directly related to Political Cynicism in its dimensions of Political Distrust, Political Hopelessness, and Political Moral Laxity, and inversely related to the dimension of Political System Change. Similarly, both dimensions of populism are directly related to Democratic Support and inversely related to Democratic Satisfaction. The findings support the notion that populist attitudes emerge in the context of distrust of the system and express an ambivalent relationship with democracy. Furthermore, the various approaches developed by the social sciences to address the populist phenomenon are discussed in terms of their strengths and limitations.

## Introduction

Populism is a symptom of a contingent relationship between political power and the need-or intention-of the masses to democratize, in a broad sense, the society. From this perspective, populism is constituted in the search for a supposed democratic constitution of power based on political decisions whose origin should be located in the masses (Villacañas, 2017; p. 17). The first aspect to highlight is that the idea of masses expressed lines before corresponds to a diffuse representation of the people, an entity that will be one of the central elements in the different approaches on the subject of populism. In this scenario, populism can be defined as a form of political action, as well as a form of government, which seeks to gain or maintain political power, based on popular adhesion and loyalty (Villacañas, 2017; p. 18).

Currently, one of the most widespread and widely used approaches in the study of populism is known as the ideational approach (Mudde, 2004; Mudde and Rovira Kaltwasser, 2017). From this approach, it is proposed that populism is a thin ideology, whose discourse confronts in a Manichean way, a representation of the people, as an idealized moral entity, before a corrupt elite, from whose actions arise the problems that the people go through. Because populism seems to adhere to other forms of full ideologies like nationalism or conservatism, among others, the idea of populism as a thin ideology is proposed (Mudde, 2004; Mudde and Rovira Kaltwasser, 2013; de La Torre, 2018; Hunger and Paxton, 2021). The aforesaid produces populisms of various political spectrums to form and manifest themselves, some of which are antagonistic to one another (Mudde and Rovira Kaltwasser, 2013; Rooduijn and Akkerman, 2017).

From an academic perspective, the ideational approach has proven useful, conceptually and methodologically, by introducing three discursive elements presented as necessary and sufficient to understand any expression of the populist phenomenon: (1) a noble people constantly exalted, (2) a corrupt elite frequently reviled, and (3) the idea that a true democracy emanates from the popular will (Mudde, 2004; Aslanidis, 2016; Mudde and Rovira Kaltwasser, 2017; Hawkins and Rovira Kaltwasser, 2017a). However, the attempt to represent populism as a thin ideology is open to a major conceptual problem, since when speaking of ideology, the notion of thinness is spurious and, in the examples proposed by those who support this approach, thin ideology entails a lack of centrality and coherence that is inconsistent with classical approaches to ideology from the social sciences (Feldman, 2013; Aslanidis, 2016).

Alternatively, to the ideational approach, the study of populism has also gone through representations of populism as a political strategy or as a discursive framework (Aslanidis, 2016). Populism as a political strategy is related to a personalistic, charismatic, and plebiscitary leadership style, through which, the populist politician builds an identitarian relationship with the people, to attain, or exercise, power based on the direct, unmediated, and non-institutionalized support of a mass of mostly unorganized followers (Weyland, 1999, 2001, 2020). On the other hand, populism as a discursive framework identifies the use of political discourse as a strategy. The contribution of this vision is that, regardless of the specificities of a particular populist project, at a general level the three constitutive elements of populism identified by Mudde and Rovira Kaltwasser (2017) are taken up again, although no longer as an ideology. The populist discursive framework might then be summed up in a discourse that asserts that a “real” democracy results from popular will, making clear the people’s moral superiority while confronting it with corrupt elites (Aslanidis, 2016).

It has already been established, in the critique of the ideational approach, that populism as a discursive framework would not constitute an elaborated and complete structure like ideologies; however, the above does not detract from the fact that it can convey coherent meanings in certain communicational situations (Moffitt and Tormey, 2014; Aslanidis, 2016; Moffitt, 2016; Ostiguy, 2020). From a psychosocial perspective, the constitutive elements of populism would comprise an intergroup dynamic where the people would act as an ingroup and the elite as an outgroup (Forgas and Crano, 2021; Stathi and Guerra, 2021). Specifically, the relationship is represented by a discursive strategy that, instead of offering rational or realistic solutions to their followers, depicts the elites as evil enemies of the people, exploiting an animosity deeply rooted in human values and needs (Forgas and Crano, 2021). Thus, populism as a framing process of political information would fulfill a cognitive function that allows people to find schemes and categories to interpret the information they receive from their environment (García Beaudoux and D'Adamo, 2007).

Gross and D'Ambrosio (2004) also point out that the framing of political information affects the emotional responses of people exposed to a message. This statement is derived from the cognitive theories of emotion from Social Psychology, from which it is proposed that evaluative and emotional responses will always be rooted in a cognitive representation of a social context, since people do not usually develop attitudes or experience emotions randomly, but rather these arise as a result of a cognitive evaluation of a given fact or phenomenon (Gross and D'Ambrosio, 2004).

In particular, populism as a discursive framework carries a political message with a particularly potent content to elicit emotional reactions, which, in line with Jerit’s (2004) proposal, would allow it to project representations that can consensually result in positive (for example, toward the moral people) or negative (for example, toward the corrupt elite) beliefs and attitudes (Aslanidis, 2016; Spruyt et al., 2021). The discursive strategy will emphasize contents that make salient the identification with the people, as opposed to the elites, to give political meaning to citizen dissatisfaction and demands (Rooduijn et al., 2016; Aslanidis, 2016; de la Torre, 2017; Marchlewska et al., 2018; Busby et al., 2019; Meléndez and Rovira Kaltwasser, 2019; Hawkins et al., 2020; Stathi and Guerra, 2021; Çakal et al., 2022). In conclusion, the populist discursive frame promises certainty and cognitive simplicity to cope with an unfavorable political situation. It also enables the development of a positive identity, a sense of moral superiority, and the promise of a collective solution to the political problem, all of which combine to appeal to its target audience (Forgas and Crano, 2021).

A complementary perspective suggests that populist beliefs and attitudes are the result of a social construction based on the interaction between political offer and citizen demands, which are framed in a context of dissatisfaction and distrust with the (liberal) democratic system (Mudde and Rovira Kaltwasser, 2017; Spruyt et al., 2021). The above has implied that populism is represented as a phenomenon in an ambivalent relationship with democracy; to some extent because both concepts are juxtaposed in the representation of a government of the people (Forgas and Crano, 2021).

Democracy can be defined in a variety of ways (see Doh, 2007; Hoffman and Graham, 2015), which impacts the ambiguity of its relationship to populism. In this context, Villacañas (2017) points out that populist methods of obtaining or exercising power must in some way be socially democratic, even if they are in conflict with liberal democracy and its values. Emphasizing this tension, Forgas and Crano (2021) indicate that in many liberal democracies there is an increase in the feeling of resentment against the elites, which would be a distinctive feature of populism. These authors add that “the rise of emotional and identity politics is replacing the old norms of rational, analytical and pragmatic decision-making, where consensus and compromise have been supplanted by implacable animosity and tribal hatreds” (Forgas and Crano, 2021; p. 2). Forgas and Crano’s (2021) observation is pertinent, but insufficient, as the authors locate the problem of populism as a threat to (liberal) democracy exclusively in the behavior of the mass-or the people-, and in populist politicians. However, they do not seem to pay attention to the contextual conditions that produce such dissatisfaction with, or distrust of, the democratic system in the mass or people (where the demand is situated), nor to the conditions in which a politician-or political movement-emerges to channel such dissatisfaction (where the offer is situated).

In this regard, authors like Inglehart and Welzel (2005) or Dargent (2013) mention the significance of the actions of-political and economic-elites in the development, improvement, or degeneration of democracy in various societies. In this vein, works by authors like Dargent (2013), Stiglitz (2013), or Cañete Alonso (2018) highlight how, over the past few decades, political and economic elites in various countries with varying degrees of democratization have taken control of State institutions and public policies through their economic and/or political power in favor of their class interests, leading to increased poverty and inequality while distorting the fundamentals of a democratic system where the common good should prevail over private interests (Cañete Alonso, 2018). As a result of this behavior of the elites, in Latin America “(t)he supports for democracy as the preferred form of government has been falling, slowly but steadily since 2010; increasing in this region the number of people who feel indifferent to the form of government adopted” (Cañete Alonso, 2018; p. 10).

The beliefs, attitudes, and political behaviors of individuals regarding how they view and exercise their citizenship, as well as how they relate to a political and social system, will be affected by the social, political, and economic characteristics of their society (Chaparro, 2018; Beramendi et al., 2020; Brussino and Alonso, 2021; Imhoff, 2021). In that sense, any discussion of the stability of a political system should focus on the quality of the exercise of governance and authority, based on compliance with transparency, procedural justice, and distributive justice that allow acceptance and trust toward the system by citizens. Then, the legitimacy of a system is constituted as part of a general political climate or culture that is fundamental for the consolidation of democracy (Tyler, 2001, 2006; Brussino and Alonso, 2021).

From the above, it could be concluded that the fundamental issue in the relationship between populism and democracy would not be the citizens’ rejection of the latter, as different opinion studies conducted at the international level have shown that, in the societies examined, the majority of people support democracy as a form of government (Inglehart and Welzel, 2005; Doh, 2007). Nevertheless, adherence to populist beliefs and attitudes would be the product of dissatisfaction-and consequent distrust-with the functioning of (liberal) democracy and the expression of its difficulties in responding to the demands of the people (Hawkins and Rovira Kaltwasser, 2017b; Hameleers and de Vreese, 2020). It is in this scenario, where demand for alternative political mechanisms to “truly” democratize society may arise in the citizenry, being there where the populist offer comes into play (see Villacañas, 2017; Meléndez, 2022).

In conclusion, it makes sense to view populist citizen demands as a sign that the (liberal) democratic system is malfunctioning. In this perspective, some populist demands would not be objectionable merely by themselves because they criticize issues that lead to distrust of the system, such as inequality, exclusion, and corruption. However, this does not mean that populism does not pose a threat to democratic regimes when it results in the violation of minority groups’ fundamental rights or the dissolution of a State’s institutional structure and power structure, among other issues that leave an authoritarian imprint (Mudde, 2004; Mudde and Rovira Kaltwasser, 2017; Villacañas, 2017).

That populism is expressed as a response, both by some politicians and the citizenry, to problematic aspects of liberal democracy (Mudde and Rovira Kaltwasser, 2017); does not mean categorically that distrust in the political system and its representatives are constituted as an exclusive or sufficient feature of it, but it is a relevant element that could predispose to its emergence (Hawkins et al., 2012; Levitsky and Ziblatt, 2018; Meléndez and Rovira Kaltwasser, 2019). Widespread distrust of the political system has been defined in social sciences as political cynicism (Miller, 1974; Siu-kai, 1992). Cynicism as an expression of discredit of the system will produce feelings of indignation, impotence, or hopelessness and a generalized perception that the political system through its actors, institutions, and norms that regulate it, lacks legitimacy for being corrupt or inefficient (Miller, 1974; Siu-kai, 1992). Moreover, as a vicious circle, cynicism will increase in contexts where there is a perceived lack of institutional legitimacy, high levels of corruption, a lack of representation of citizen interests by politicians, perceived lack of distributive and procedural justice, among others (Miller, 1974; Siu-kai, 1992; Beramendi, 2014).

The consequences of political cynicism are considered potentially dangerous for the development of a society, as it mitigates civic and democratic values and attitudes, and tends to reduce citizen participation in the political sphere, as a result of hopelessness or disinterest in public affairs (Patterson, 2002; Chaparro, 2018) and increases citizens’ adherence to authoritarian or populist political positions as forms of opposition to the political system and what it has traditionally represented (Bélanger and Aarts, 2006; Levitsky and Ziblatt, 2018; Çakal et al., 2022). Espinosa et al. (2022c) find in a recent study in 11 Ibero-American countries, that the elements that constitute political cynicism goes beyond political distrust toward the system in general, so it is important to extend its understanding to the perception of corruption present in the system as a specific element on which distrust develops. Likewise, the authors refer that it is important to include in the description of political cynicism the elements of Political Moral Laxity and the Perception of the need for change in the system as possible outcomes of distrust, to the extent that they explain the types of representation and political participation demanded by the citizenry (Espinosa et al., 2022a,c).

The aforementioned suggests that a populist discursive framework could develop from the expressed distrust of the system, where society and its citizens are portrayed in the two groups mentioned above: “the moral people” vs. “the corrupt elites.” (Aslanidis, 2016; Mudde and Rovira Kaltwasser, 2017; Çakal et al., 2022). As was previously observed, the previous classification places different identity processes at the core of populist discourse, which is how populist promoting agents attempt to persuade people to support their political objectives (Marchlewska et al., 2018; Stathi and Guerra, 2021; Çakal et al., 2022). This produces an interesting paradox since, on the one hand, identity affiliations to processes of populist nature establish a position where malfunctioning and corruption in the system are perceived as alien to those who adhere to the populist cause. Thus, for example, rhetorics about corruption frame a corrupt and unreliable “other” to whom are attributed, through denunciation, the difficulties that members of the ingroup (the moral people) would be facing (Mudde and Rovira Kaltwasser, 2017; Çakal et al., 2022). However, on the other hand, processes of political, economic, and social crisis where populism emerges, are also often framed by institutional weakening and corruption, which has produced in the citizenry a certain tolerance and acceptance of these problems at the individual and institutional level (see Quiroz, 2013; Levitsky and Ziblatt, 2018).

In Latin America, there is an emergence of various populist and authoritarian political movements, both on the ideological left (for example, Chavismo in Venezuela) and on the ideological right (for example, Bolsonarismo in Brazil), where the hegemonic discourses at the base of such movements refer to a refoundation of the political system with narratives that confront the so-called good citizens-or the moral people-against the corrupt elite (Salinero, 2015; Mudde and Rovira Kaltwasser, 2017; Levitsky and Ziblatt, 2018; Meléndez, 2022). In this scenario, a new paradox arises, since in societies such as those described, a tendency to accept and tolerance toward corrupt practices is often appreciated, incorporating certain moral laxity (Espinosa et al., 2022a,c), which is a potentially dangerous component of political cynicism. Political moral laxity is a condition that is typical of many politicians and political movements linked to discourses of a populist nature, specifically when narratives about the election of candidates with a history or suspicion of corruption are acceptable as long as “they steal but do work” or “they benefit me and my groups,” opening the door to prebendary and clientelist political strategies in society (Quiroz, 2013; Sautu, 2014; Schmitz and Espinosa, 2015; Janos et al., 2018). In this regard, López-López et al. (2016, 2017), found in several studies developed in Colombia that attitudes toward corruption do not depend exclusively on the corrupt act or its consequences; rather, the levels of tolerance toward corruption will be related to a set of characteristics of the corrupt actor, from which behavior will be problematized as corruption or not, which will depend on the political and identity affinity that citizens have with whoever performs the corrupt act. This is consistent with the observations of Dargent (2013), who introduces the notion of precarious democrats, attributing it to political elites-and could be extended to their followers-who express different levels of adherence and defense of democracy according to their convenience. They express a strong detachment to it when they are in power and vindicate it when they have to play an opposing role to other politicians in power, which they usually accuse of being undemocratic.

Despite the extensive reference to the concepts of political cynicism and populism in the social sciences, the empirical approach to them, and their relationship, is scarce in social psychology (Feldman, 2013; Çakal et al., 2022) so the present study opens a line of research little explored and original, locally and internationally. The scenario described becomes more interesting because, in the Peruvian context, there has been much discussion about populism and distrust (political cynicism), without going into detail on those elements of Peruvian political culture that explain how the relationship between the two occurs and how it is experienced by the country’s citizens, as well as how these phenomena are related to attitudes and satisfaction with democracy, in a scenario of democratic precariousness (Dargent, 2013). Regarding the latter, a recent study by Chaparro et al. (2022), which analyzes which variables predict populist attitudes in Chile, Colombia, and Peru, finds that populist demand at the general level is directly explained by (1) a positive attitude toward pluralism-as demand for social inclusion-, (2) the self-perception of relative deprivation-as a subjective expression of lack of distributive justice-, (3) the political cynicism in its dimensions of generalized distrust, need for change and the perception of corruption; and (4) the perception of poor democratic functioning. While populist attitudes will be inversely related to greater political moral laxity. In other words, populist attitudes, as a citizen demands in the studied countries, are a covariation of different elements perceived as flaws in the functioning of the democratic system, which need to be modified.

On the other hand, Espinosa et al. (2022b), in a qualitative study, investigate how populism and democracy are socially represented in a sample of Peruvian citizens from different regions of the country. The originality of this approach is that it portrays the representation of populism and democracy from ordinary citizens, and not in descriptions coming from academia as those mentioned above. Beginning with the representation of democracy obtained in the results, it can be seen that this is semantically poor, being associated mainly with electoral behavior and, to a lesser extent, with the representation of an exercise of rights and freedoms or the idea of a government of the people-or the majority-; while populism is represented as a set of strategies to influence the political behavior-mostly electoral behavior-of the people, exploiting their basic needs. From the above, a negative representation of populism emerges, where the masses that express adherence to populist strategies are described as manipulable entities due to their ignorance or disinterest in public affairs. The notion of populism also includes a negative representation of politicians in general-including populist politicians who, through various usually unscrupulous strategies, try to win popular favor. In sum, populism is seen as a negative phenomenon, where the corrupt elites are the politicians-and on very rare occasions the economic elites are mentioned-, where there is no representation of a moral people, but of a people that can be manipulated because of their ignorance, and where, if anything, the only positive aspect is that it is a symptom of issues that need to be resolved within the system (Espinosa et al., 2022b). The above implies that, from an approach of social representations, populism for the citizenry differs from the popular (see Aslanidis, 2016), and its definition is tinged with a negative nuance with which there is no identification. That is, populism is not situated in oneself, but in an “other” that can be manipulated because of its ignorance or because it obtains some benefit from clientelist and prebendary strategies, which implies a negative perception of the populist offer.

The context of the current study is interesting because various analyses of the political reality in Peru indicate a tendency among citizens to view the political system negatively, which has led to mistrust and indifference in public affairs (Chaparro, 2018; Janos et al., 2018). The negative perception of the system appears to be anchored in a variety of phenomena, such as corruption, which appears to be an endemic problem in the country (Quiroz, 2013) and whose representation has gained public attention as various scandals involving political actors from various state powers and political parties have been exposed in the media (Proética, 2019). Recently, the described scenario has also included processes of political polarization promoted by some actors and political groups with the intention of obtaining power quotas at the expense of weakening institutions and further undermining the country’s democratic political system (Dargent, 2013). The above has gotten worse with the arrival of the COVID-19 pandemic in Peru, highlighting the Peruvian State’s inability to deal with a complex health situation like the one described, and making Peru one of the countries with the worst emergency care performance, resulting in a large number of deaths and increased poverty in the country (Villarán et al., 2021). Thus, in the period between 2021 and 2022, when this study was conducted, and in the face of new presidential elections, various political groups and actors emerge on the electoral scene with offers that, discursively and strategically, could fit the definitions of populism (Meléndez, 2022). From the foregoing, it is clear that there is a high prevalence of political cynicism in Peru (Chaparro, 2018; Espinosa et al., 2022c) as well as a large presence of a diverse populist offer by several actors and political groups attempting to respond to citizen demand for representation (Meléndez, 2022). Despite the fact that the social sciences have proposed systematic relationships between these two political processes and how they can affect democracy, empirical evidence has been limited.

Based on what has been described so far, the present proposal has the general objective of describing and analyzing the beliefs and attitudes toward populism, at two analytical levels (1) populist attitudes comprising populist demand (Akkerman et al., 2014) and (2) the perception of the populist offer (Espinosa et al., 2022b), and their relationships with (3) political distrust expressed in the dimensions of political cynicism (Espinosa et al., 2022c) and (4) attitudes toward democracy in the indicators of support and satisfaction with this system of government.

## Materials and methods

### Participants

The sample consisted of 391 people with an age range between 18 and 82 years (*M* = 34.39, *SD* = 15.25), of whom 56.3% were men and 41.4% were women (the remaining percentage were people who were non-binary or preferred not to state their sex). Concerning self-perceived socioeconomic level (SES) the majority of participants represented themselves as middle level (49.6%), a second group considered themselves to be lower-middle level (26.9%) and in third place was the group that considered themselves to be upper-middle level (13.8%). The groups with the lowest frequency were the high and low-level groups. About the region of residence, the majority of participants were from Lima (50.7%) while the second largest group was from San Martín (23.3%); the rest of the participants were distributed homogeneously and with small percentages in the rest of the regions of the country.

Additionally, we asked about other variables that help to contextualize the socio-political characteristics of the participants such as (1) their level of interest in politics, (2) their political orientation in the left–right continuum, and, finally, (3) their attitudes toward the economic model change in the country. It was evident that there was a tendency to report interest in politics (57.1%), a slight tendency to self-position themselves in the ideological center (38.6%) and, in the same way, there is a slight tendency of the participants to seek to change the economic model (45%).

### Ethical considerations

Due to its characteristics, the present study does not involve sensitive contents that could generate any risk to the physical or psychological health of the participants. However, to comply with the requirements and ethical considerations of a project of this nature, participants were presented with the terms of the informed consent, which they had to read and accept to agree to answer the study questionnaire. The informed consent explained the general objective of the study and the conditions of anonymity and confidentiality under which the information obtained would be managed. Participants were also informed of the academic nature of the study and that the information obtained would be used exclusively for this purpose. In addition, it was emphasized that participation was voluntary and that they could stop answering the questions in the questionnaire at any time if they so wished. Finally, they were asked to leave an e-mail address if they wished to receive information on the final results of this project.

### Measurement

#### Political cynicism scale

The version adapted by Espinosa et al. (2022c) from the original version constructed by Janos et al. (2018) was used. The instrument consists of 12 items on a Likert scale ranging from 1 (Strongly disagree) to 4 (Strongly agree). A CFA was performed in the original study to evaluate the factorial structure of the scale and a final 4-factor solution was found with a good fit, *χ*^2^/df = 9.967, CFI = 0.960, NFI = 0.956, RMSEA = 0.061, 90% CI [0.054, 0.069] (Espinosa et al., 2022c). The 4 factors found were: (1) Political Distrust, which evidences a negative and incredulous view toward political institutions and authorities, as well as politics in general (*α* = 0.78 and *ω* = 0.80); (2) Political Moral Laxity, which shows favorable attitudes toward maintaining a corrupt and inefficient political system but oriented to satisfy the needs of certain groups, without taking into account the damage or harm toward other sectors or toward society itself (*α* = 0.61 and *ω* = 0.63); (3) Political System Change, which indicates that it is necessary to make modifications to the current system to achieve improvements in society (*α* = 0.78 and *ω* = 0.79); and (4) Political Hopelessness, which evidences a pessimistic view of politics in general due to the widespread corruption in the system (*α* = 0.43 and *ω* = 0.58). Although the alpha coefficient is low, this could be happening because the tau equivalence assumption is not met. This could be leading to an overall underestimation of reliability and, therefore, to a lower than expected score (McNeish, 2018; Hayes and Coutts, 2020). Because of this, the omega coefficient (which does not require this assumption) is used and a higher value than the previous one is observed. This new coefficient has acceptable levels of internal consistency for statistical inferences, according to the criteria of Mezulis et al. (2004).

#### Attitudes toward democracy

Two items were used specifically focused on evaluating both the support (“In general I believe that democracy is the best system of government”) and the satisfaction (“I am satisfied with the functioning of democracy in Peru”) of the participants toward the democratic system of government in Peru. For both items, a Likert scale ranging from 1 (Strongly disagree) to 5 (Strongly agree) was used. Both items were analyzed independently, taking into account that each of them explains different elements of the country’s social reality. These items do not have psychometric information but have been used previously in similar studies such as those of Chaparro et al. (2022) and Rovira Kaltwasser and Van Hauwaert (2020) demonstrating a correct performance.

#### The scale of populist attitudes

The scale developed by Van Hauwaert et al. (2016) was used, which consists of 8 items evaluated on a Likert scale from 1 (Strongly Disagree) to 5 (Strongly Agree) that measure populist attitudes based on previous studies such as Hawkins et al. (2012) and Akkerman et al. (2014). Additionally, 4 items were added that evaluated additional aspects of the representation of populism, not represented in the original scale previously cited (see Espinosa et al., 2022b), this decision to incorporate new items more focused on the perception of the populist offer also helped to improve the validity of the instrument. In that sense, an Exploratory Factor Analysis (EFA) was performed to know the underlying structure and an optimal sample fit was found (KMO = 0.870, *χ*^2^ = 1859.85, *p* < 0.001) with two factors explaining 48.34% of the total variance. The first factor was composed of all the items of the original Van Hauwaert et al. (2016) scale plus one of the items added for this study (in total there were 9 items) and was labeled “Populist Demand.” The second factor was composed of 3 items and was called “Perception of the Populist Offer,” which includes both a representation and a negative evaluation of populism. The reliability of both factors for the present study was good (Populist Demand: *α* = 0.83 and *ω* = 0.83; Perception of the Populist Offer: *α* = 0.86 and *ω* = 0.81).

### Procedure

The application protocol was developed in the *Qualtrics* survey platform where all the instruments were digitized. The sampling of participants was non-probabilistic and was carried out through the use of social media (Facebook, Twitter, etc.) and the snowball technique to obtain the desired cases. At the beginning of filling out the scales, there was an informed consent. Subsequently, the instruments were answered in the following order: sociodemographic data (including certain questions on socio-political characteristics), political cynicism, populist attitudes, and finally attitudes toward democracy. The fieldwork was carried out between December 2021 and January 2022. Once the questionnaire was closed, the database was exported to the statistical software and the corresponding analysis began.

### Data analysis

The data were analyzed with IBM SPSS statistical software version 27. Initially, the database was cleaned to check for outliers and missing cases. Based on the latter, the amount of missing data did not exceed 5% per observed variable, so it could be considered as part of a random and non-systematic process (Ho, 2013). In any case, some cases that did not meet at least 90% of completed responses were eliminated, generating the final sample of 391 participants. With the base already cleaned, a normality analysis was performed using the Shapiro–Wilk test and the skewness and kurtosis statistics. It was found that the univariate normality assumption was met for all variables. A descriptive analysis was then performed for all the dimensions of the study variables. Finally, and as the main procedure, correlation analysis was performed using Cohen’s criteria for the magnitude of the effect size, and also regression analysis using the stepwise method, where the dimensions obtained from the populist attitudes scale were treated as dependent variables. The stepwise method is the step-by-step iterative construction of a regression model involving automatic selection of independent variables (different from hierarchical regression). This method was used to more easily identify those variables that should be included in the model and those that could be excluded based on a series of F-tests and t-tests.

## Results

### Descriptive analysis

As an initial part of the analysis, descriptive statistics were performed for the four dimensions of Political Cynicism, Attitudes Toward Democracy, and the two factors of the Populist Attitudes Scale. The results can be seen in Table 1.

**Table 1:** Descriptive statistics of study variables.

Variable	*n*	*M*	DE	95%CI
Political cynicism				
Political distrust	391	3.09	1.00	[2.99, 3.19]
Political moral laxity	391	3.92	0.73	[3.85, 4.00]
Political system change	390	1.91	0.83	[1.82, 1.99]
Political hopelessness	391	3.69	0.77	[3.61, 3.76]
Attitudes toward democracy				
Democracy support	380	3.87	1.01	[3.77, 3.98]
Satisfaction with democracy	387	2.16	0.94	[2.07, 2.26]
Populist attitudes				
Populist demand	391	3.72	0.64	[3.66, 3.78]
Perception of the populist offer	390	4.30	0.74	[4.22, 4.37]
Socio-political characteristics				
Interest in politics	389	3.62	1.21	[3.50, 3.74]
Left–right political orientation	390	3.00	1.17	[2.88, 3.12]
Economic model change	390	3.24	1.39	[3.10, 3.38]

Concerning the dimensions of the Political Cynicism scale, it can be seen that almost all the scores are above the midpoint of the response scale (2.5) and even reach almost the maximum score. The only exception is the factor of Political System Change, which is below that level. Regarding attitudes toward democracy, there is a high level of Support for this system of government (being above the midpoint), however, the level of Satisfaction with Democracy is somewhat low (being below the midpoint). On the other hand, in the case of both dimensions of populist attitudes (demand and perception of offer) participants demonstrate high levels in these variables (above the midpoint), highlighting that Populist Demand comprises a favorable valuation of the empowerment of the people in political decision-making, while Perception of Populist Offer is semantically represented negatively. Finally, the participants declared a medium to high interest in politics (above the midpoint), a centrist ideological position, that is, little defined toward the extremes of the left–right continuum, and a medium to high Disposition toward the change of the economic model (above the midpoint).

### Relationships between political cynicism, attitudes toward democracy, and populist attitudes

In response to the main objective, several correlation analyses were carried out between the different variables of the study. The results of these relationships can be seen in Table 2.

**Table 2:** Correlations between the dimensions of political cynicism, attitudes toward democracy, socio-political characteristics and populism.

	1	2	3	4	5	6	7	8	9	10	11
1. Political distrust	1										
2. Political moral laxity	0.137^**^	1									
3. Political system change	0.305^**^	−0.106^*^	1								
4. Political hopelessness	0.355^**^	0.178^**^	0.045	1							
5. Democracy support	−0.051	0.013	−0.185^**^	0.058	1						
6. Satisfaction with democracy	−0.077	−0.097	0.167^**^	−0.102^*^	0.176^**^	1					
7. Populist demand	0.345^**^	0.353^**^	−0.104^*^	0.286^**^	0.177^**^	−0.036	1				
8. Perception of the populist offer	0.285^**^	0.222^**^	−0.179^**^	0.342^**^	0.163^**^	−0.149^**^	0.487^**^	1			
9. Politics interest	−0.293**	0.014**	−0.219	−0.035	−0.056	−0.201**	−0.095	−0.064	1		
10. Left–right political orientation	0.119*	−0.161**	0.249**	−0.054	0.036	0.163*	−0.301**	−0.035	−0.192**	1	
11. Economic model change	0.011	0.166**	−0.083	−0.017	0.001	−0.054	0.391**	−0.090	0.052	−0.368**	1

*p < 0.05; **p < 0.01.

The main results of these correlations are summarized as follows: (1) the Political System Change dimension is significantly and inversely related to Democracy Support and directly related to Satisfaction with Democracy. In addition, Political Hopelessness is negatively associated with Satisfaction with Democracy; (2) The dimensions of Political Distrust, Political Hopelessness, and Political Moral Laxity are positively associated with both dimensions of populist attitudes (Populist Demand and Perception of the Populist Offer), while the dimension of Political System Change is, in both cases, inversely associated. Finally, (3), it can be seen that Populist Demand and Offer Perception correlate directly with Democracy Support. Likewise, Perception of the Populist Offer is inversely related to Satisfaction with Democracy.

### Regression analysis between political cynicism, attitudes toward democracy, socio-political characteristics, and populist attitudes

To understand the relationships between the variables, several multiple linear regressions were performed using the stepwise method, with political cynicism and attitudes toward democracy as predictor variables and the dimensions of populism as the criterion variable. Additionally, certain socio-political characteristics were also incorporated as part of the analysis, such as whether they are in favor or against the change in the economic model (“Economic Model Change”) and its political orientation (“Left–Right Political Orientation”). Sociodemographic variables such as age, sex or socioeconomic level were not taken into account as part of the regression model or as control variables, because there was no association between these variables and the study variables mentioned above.

For the first dimension, a significant model was obtained that explains 41.2% of the variance of the Populist Demand dimension, *F*(6,347) = 42.25, *p* < 0.001. Specifically, all dimensions of Political Cynicism, except for Political System Change would be predictive of Populist Demand. Of the dimensions of attitudes toward democracy, only Democracy Support would be predicting Populist Demand. Economic Model Change also directly predicts Populist Demand. Finally, ideological orientation has an inverse relationship with Populist Demand, that is, people who are more to the left of the ideological continuum tend to score higher on Populist Demand (see Table 3). The following variables were excluded from the model because they were not significant: Satisfaction with Democracy, Political System Change and Interest in Politics.

**Table 3:** Multiple linear regression of political cynicism, attitudes toward democracy and socio-political characteristics (IV) on the populist demand dimension (DV).

	Estimate	*SE*	95% IC		*p*
			*LL*	*UL*	
Intercept	1.39	0.23	0.95	1.85	<0.001
Political distrust	0.18	0.02	0.13	0.24	<0.001
Economic model change	0.13	0.02	0.09	0.17	<0.001
Political moral laxity	0.21	0.04	0.14	0.29	<0.001
Left–right political orientation	−0.12	0.03	−0.17	−0.07	<0.001
Democracy support	0.15	0.03	0.07	0.17	<0.001
Political hopelessness	0.11	0.04	0.04	0.18	0.003

For the second dimension, a significant model was obtained that explains 22.7% of the variance of the Perception of the Populist Offer dimension, *F*(6,347) = 18.31, *p* < 0.001. About the coefficients, something very similar happens as in the previous model; all the dimensions of Political Cynicism would be directly predicting the Perception of the Populist Offer, except for the Political System Change which does so inversely. Likewise, Democracy Support directly and a Disposition to Economic Model Change inversely would also be part of the statistical model (see Table 4). The following variables were excluded from the model because they were not significant: Satisfaction with Democracy, Left–Right Political Orientation and Interest in Politics.

**Table 4:** Multiple linear regression of political cynicism, attitudes toward democracy and socio-political characteristics (IV) on the perception populism offer dimension (DV).

	Estimate	*SE*	95% IC		*p*
			*LL*	*UL*	
Intercept	2.54	0.29	1.97	3.10	<0.001
Political hopelessness	0.23	0.05	0.13	0.32	<0.001
Political system change	−0.21	0.05	−0.30	−0.12	<0.001
Political distrust	0.19	0.04	0.11	0.26	<0.001
Economic model change	−0.07	0.03	−0.12	−0.02	0.005
Political moral laxity	0.16	0.05	0.06	0.26	0.001
Democracy support	0.09	0.03	0.02	0.16	0.009

### Path analysis of the predictors of populist attitudes

**Figure 1:**
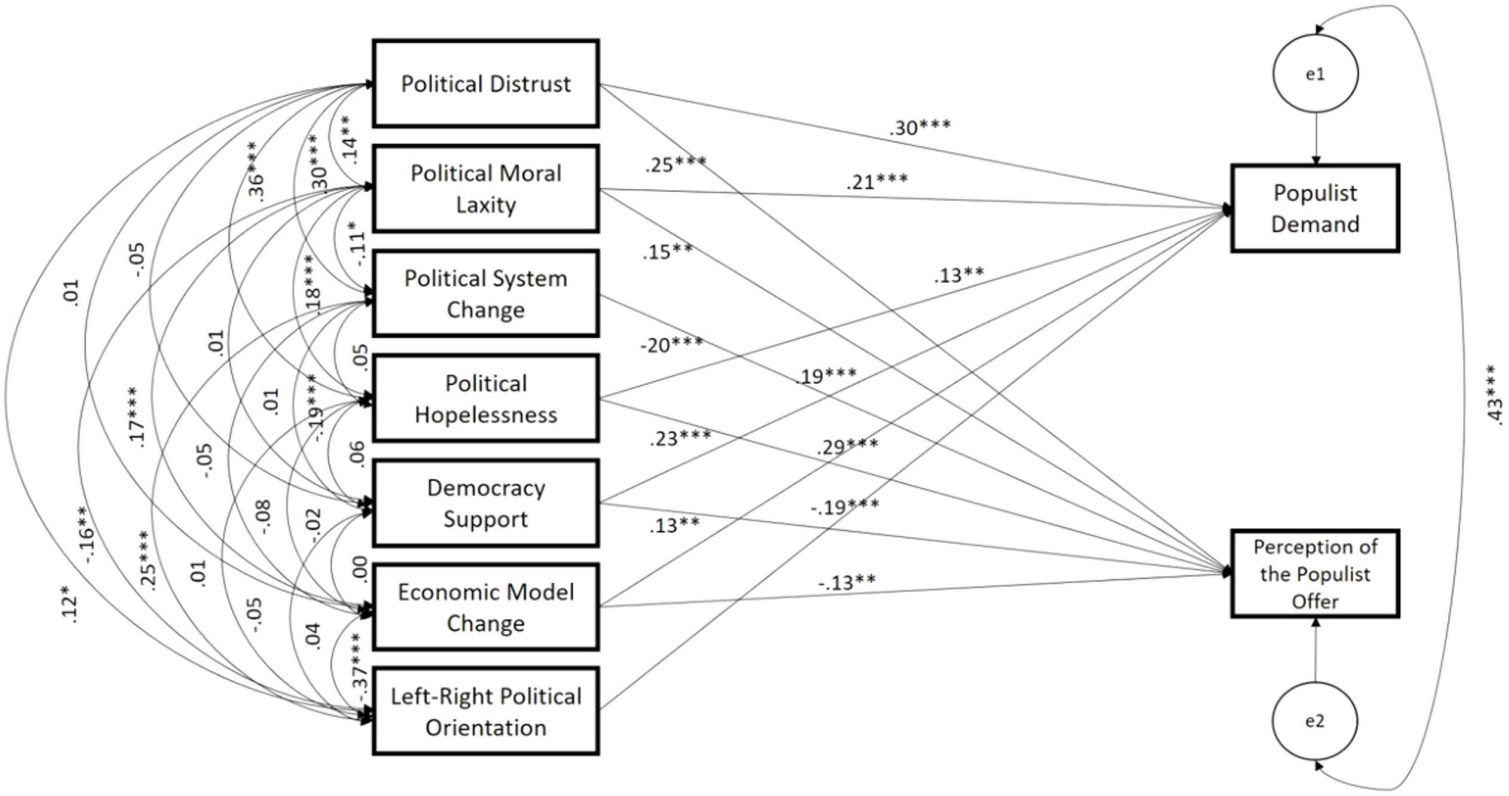
Path analysis of the proposed model. *p < 0.05; **p < 0.01; ***p < 0.001.

Based on the obtained results, a path analysis was processed to observe in an integrated model how the Populist Demand and the Perception of the Populist Offer as exogenous variables were related to the dimensions of Political Cynicism, the dimensions of Attitudes toward Democracy, and the Socio-Political Characteristics of the participants as endogenous variables (see Figure 1). The model obtained shows a good fit according to the following indices: *χ*^2^/df = 2.364, CFI = 0.995, NFI = 0.993, RMSEA = 0.059, 90% CI [0.000; 0.130].

## Discussion

On a descriptive level, it is clear that the sample studied has a high level of Political Cynicism, —for example, the participants distrust their political system, perceive it to be corrupt, and feel hopeless about it. Simultaneously, it is a sample with high levels of Political Moral Laxity, which means that in political decision making they tend to prioritize private and individual interests before the public good (Espinosa et al., 2022a). As a whole, the dimensions of Political Cynicism and, more specifically, its component of Political Moral Laxity builds a favorable path for a political offer that, far from solving the underlying problems of inclusion, inequality, or corruption, systematically becomes the “lesser evil” or a political offer constituted by those who “steal but [at least] do work” (Espinosa et al., 2022a; Meléndez, 2022). The above has a bearing on the fact that the [Negative] Perception of the Populist Offer is high in the sample. Paradoxically, the Political System Change does not stand out, with which distrust, in general, is constituted as a central element in the deterioration of quality, both in the populist offer perception and in the demand for political representation (Espinosa et al., 2022c).

The scenario of generalized distrust of the system produces a complex relationship with a democracy made precarious by the political behavior of the elites (Dargent, 2013), because although participants are more inclined to support it as a political system, they are dissatisfied with its functioning. Despite the fact that support for democracy is a consistent phenomenon at the international level (Inglehart and Welzel, 2005), there is not necessarily a consensus on liberal democracy, although there is support for more limited representations of democracy, based on electoral behavior or the idea of a government of the people, which are expressions of social democratization that reflect popular demands for economic or social inclusion, but do not necessarily incorporate respect for pluralist or libertarian values (Espinosa et al., 2022b).

Regarding attitudes, which comprise the Populist Demand (Van Hauwaert et al., 2016), there is a medium-high tendency to support ideas that democracy should emanate from the people; however, when the questions are channeled toward the Perception of the Populist Offer, this is viewed negatively and as part of the critical view of populist politics and politicians in the country (Espinosa et al., 2022b; Meléndez, 2022). The semantic discrepancy observed in the indicators of populist attitudes, Populist Demand with a more positive nuance and Perception of the Populist Offer (by those who demand) with a more negative nuance, can be explained by the fact that, in the classic measurement of populist attitudes, the term “populism” has not been made explicit, while in the items added to the scale that evaluate the Perception of the Populist Offer, this term is presented explicitly. The above introduces a critical methodological aspect about some scales of populist attitudes since there is an important semantic and evaluative distinction between populism, which is what is intended to be measured, and the popular, which is ultimately what is measured (Aslanidis, 2016).

At the correlational level, populist attitudes are associated with all the indicators of Political Cynicism evaluated. As previously argued, although it is not a sufficient condition, widespread distrust of a system can trigger populist demands in a society (Hawkins et al., 2012; Levitsky and Ziblatt, 2018; Meléndez and Rovira Kaltwasser, 2019). From the perspective of a citizenry that distrusts, is hopeless, has developed morally lax attitudes, and expresses no interest in changing the system, greater adherence to the idea of empowerment of the people is appreciated. This implies a paradoxical political process where the populist demand is anchored in distrust and dissatisfaction with the system, but where, in the end, there is no expectation of substantive change either. It is something like finding oneself in a country of citizens dissatisfied with the system, but only in those aspects that are not functional to their ingroup or class interests (Dargent, 2013; Meléndez, 2022). In addition to the above, it can be seen that support for democracy in a distrustful and politically hopeless citizenry, with a disposition to moral laxity and little interest in changing things, could be related to a diffuse or poor representation of democracy (Espinosa et al., 2022b); That is, support for democracy in this sample, does not necessarily coincide, and may even enter into tension, with liberal democracy, and its principles of respect for plurality and individual freedoms, or with expressions of republican democracy that seek to ensure institutional strengthening and balance of state powers (Mudde and Rovira Kaltwasser, 2017; Villacañas, 2017; Forgas and Crano, 2021).

Meléndez (2022) mentions that Peruvian society, despite its social and political diversity, is systemically populist, and its members act accordingly, regardless of the group to which they belong. Thus, populism is constituted as a way of doing politics generalizable to almost all social and political strata of Peruvian society, although as mentioned by Espinosa et al. (2022b), none of them is directly identified with the populist category.

In the sample, although the populist demand is not associated with the need for political change, it is associated with the need to discuss the change of the economic model, which seems to be related to the perceived-and existing-problems of distributive justice in the country (Chaparro et al., 2022). The latter is also linked to the economic *anti-establishment* discourse in Peru, which has been mostly claimed by different factions of the Peruvian left, observing in this study a relationship between left-wing ideological positioning and attitudes that comprise a populist demand. In contrast, the study sample shows that the more right-wing a participant is, the less populist attitudes and the less *anti-establishment* demands at the economic or political level.

An important element, and returning to a limitation previously alluded to in the interpretation of the so-called populist attitudes, is the use of the scale of Van Hauwaert et al. (2016), whose statements do not show (1) explicit references to the concept of populism, while, (2) the references to elites, without explicitly using this term, focus on politicians and not on other power groups. In contrast, in the qualitative study developed by Espinosa et al. (2022b) in Peru, the representations of populism carry a critical and negative perception of the populist offer, when the concept “populism” is made explicit. Likewise, politicians in general, and populist politicians in particular, are negatively referred to as part of the problems derived from this political phenomenon. The above, as has been discussed, causes people not to identify themselves as populists.

As seen, the negative perception of the populist offer is consistently associated with all the evaluated elements of political cynicism. On the one hand, the perception of populism is directly related to greater distrust, greater hopelessness due to perceived corruption, and greater justification of political morally lax attitudes. Likewise, it is related to a lower need for change in the political system; which, although it seems paradoxical, could be explained by the fact that there is a generalized perception of systemic malfunctioning, where transgression practices and non-civic behaviors have been reinforced in citizens, which, although perceived negatively, can be seen as functional and therefore, ultimately acceptable (Quiroz, 2013; Chaparro, 2018; Beramendi et al., 2020).

An additional element of interest is that the greater the negative perception of the populist offer, the less satisfied people are with democracy, although they support it as a system of government, which paradoxically implies that, despite the malfunctioning of the system and a negative perception of the populist offer as a way of doing politics in the country (Espinosa et al., 2022b; Meléndez, 2022), people persist in expressing their support for democracy as a system of government (Inglehart and Welzel, 2005).

The direct relationship between the negative perception of the populist offer with the populist demand seems to be explained by the fact that, in the face of a bad political offer, popular empowerment is necessary as a way to democratize the system and its shortcomings, which implies a greater adherence to populist attitudes focused on demand because as has been said, these have been measured through a scale that, does not explicitly refer to populism, and does refer to the popular (Aslanidis, 2016).

The integrated results seem to represent two aspects of populism that are overlapping each other. The covariation of demand and perception of the populist offer would be explained as a diffuse need to democratize society and a negative perception of political offer-mostly populist-as a way to do so.

The results of the populist attitude scale, where demand is located, are associated with the need to change the economic model, with a greater left-wing ideological orientation and with greater support for democracy; at the same time, which is related to distrust and hopelessness toward the system and a prevalence of greater political moral laxity, this last point is a possible result of the so-called “tribal” elements in populist attitudes, and which are characterized by interested support for an ingroup, even at the cost of harming other outgroups with which life in society is shared (Forgas and Crano, 2021). In other words, populist attitudes finally seem to support the idea of social democracy, not necessarily pluralist, libertarian, or republicanist (Villacañas, 2017; Meléndez, 2022).

On the other hand, the perceived populist offer, although negative, is not related to an intention to change the system, which would imply an impoverishment of social capital through the acceptance of a system that is unreliable, corrupt, and in which it is acceptable to make morally lax political decisions that may have a positive effect on private or ingroup interests while affecting the common good. The above, as expected, could comprise a gradual weakening of civic and democratic values (Cañete Alonso, 2018; Espinosa et al., 2022a).

In sum, the results are consistent with a negative representation of populist offer, although there seems to be functional habituation toward it. On the other hand, the demand seems to seek to democratize, albeit in a limited way, society by taking power away from the politicians who are perceived more negatively, but putting in their place others who are less bad; which would be at the base of a recurrent phenomenon in Peruvian political life, which consists of voting for the “lesser evil” (Espinosa, 2008). The apparent inconsistency of some results seems to be explained by the idea that the way of doing politics in Peru can be understood as totally populist (Meléndez, 2022). Thus, in light of the political results of the last two decades, populism comprises a set of strategies established to vindicate an anti-establishment discourse, about which there is little expectation and, therefore, has a limited capacity for the constant mobilization of the citizenry to seek a change in the system.

Finally, while it is true that the study proposes an interesting discussion on populism, it is not exempt from limitations. Thus, it is considered that the sample, although large, lacks national representativeness. It is suggested that the sample should be more diverse in terms of place of residence and political views. In addition, the way of measuring democracy may not adequately represent the construct of support and satisfaction by only evaluating it with two items, despite the fact that these items function correctly in previous studies. It is proposed to use other scales of attitudes toward democracy that can better reflect the manifestations of the construct. Finally, not as a limitation in itself, but an additional validation of the populist attitudes scale should be carried out. It would be pertinent to carry out a CFA in order to confirm the factorial structure found in this study and to have more evidence of the validity of this construct since only an exploratory look at the structure of the test was evidenced.

## Data availability statement

The raw data supporting the conclusions of this article will be made available by the authors, without undue reservation.

## Ethics statement

Ethical review and approval was not required for the study on human participants in accordance with the local legislation and institutional requirements. The patients/participants provided their written informed consent to participate in this study.

## Author contributions

AE, EJ, and MP: theoretical proposal and methodological design, data analysis, and final report writing. JJ: methodological design and final report writing. HC: theoretical proposal and final report writing. All authors contributed to the article and approved the submitted version.

## Funding

This work was supported by the Vicerrectorado de Investigación de la Pontificia Universidad Católica del Perú (VRI-PUCP) FAI-0048-2022.

## Conflict of interest

The authors declare that the research was conducted in the absence of any commercial or financial relationships that could be construed as a potential conflict of interest.

## Publisher’s note

All claims expressed in this article are solely those of the authors and do not necessarily represent those of their affiliated organizations, or those of the publisher, the editors and the reviewers. Any product that may be evaluated in this article, or claim that may be made by its manufacturer, is not guaranteed or endorsed by the publisher.
